# Supplementary material for: Structural insights into the gating of DNA passage by the topoisomerase II DNA-gate
Source: Nat Commun. 2018 Aug 6;9:3085. doi: 10.1038/s41467-018-05406-y (PMC6078968; doi:10.1038/s41467-018-05406-y)
Supplement: Supplementary file 1 — Supplementary Information [file 41467_2018_5406_MOESM1_ESM.pdf]

Structural Insights into the Gating of DNA Passage by the Topoisomerase II DNA-Gate

Chen et al.

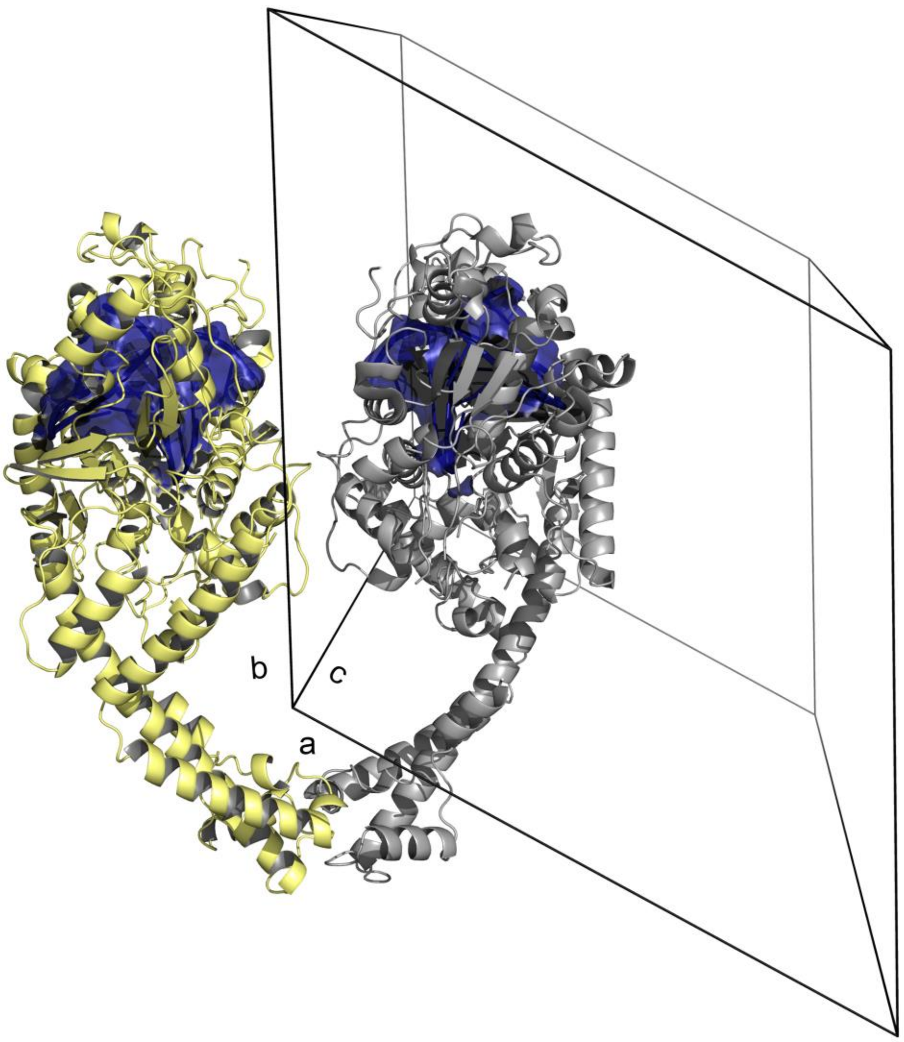

Supplementary Figure 1. Construction of a functionally relevant Top2 homodimer. The asymmetric unit is composed of one Top2 monomer (gray) bound to a 9-bp DNA duplex (blue). Given that Top2 functions as a homodimer and that the dimeric architecture of the cleavage core is mainly maintained by the C-terminal dimerization interface, the biologically relevant interacting partner (yellow) can be readily identified to construct a functional Top2 homodimer. In this case, the molecular 2-fold axis of Top2 coincides with the crystallographic dyad parallel to the unit cell b-axis.

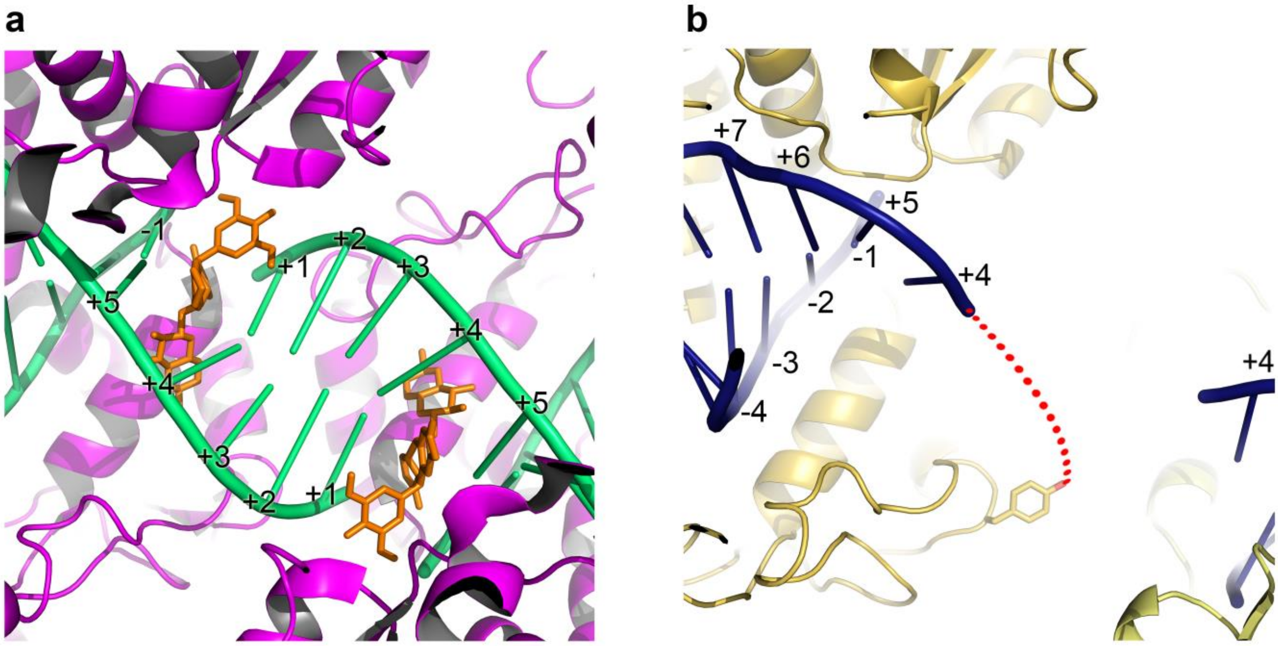

Supplementary Figure 2. The first three nucleotides of the protein-linked cohesive end become fully disordered upon DNA-gate opening. (**a**) When the DNA-gate is in the closed conformation[1] (PDBid: 3QX3), the protein-linked cohesive ends are structurally ordered and form Watson-Crick base pairing. (**b**) Upon DNA-gate opening, the first three nucleotides (+1~+3 positions) become fully disordered, likely due to the lack of interactions with nearby protein residues and the loss of Watson-Crick base pairing with the complementary strand.

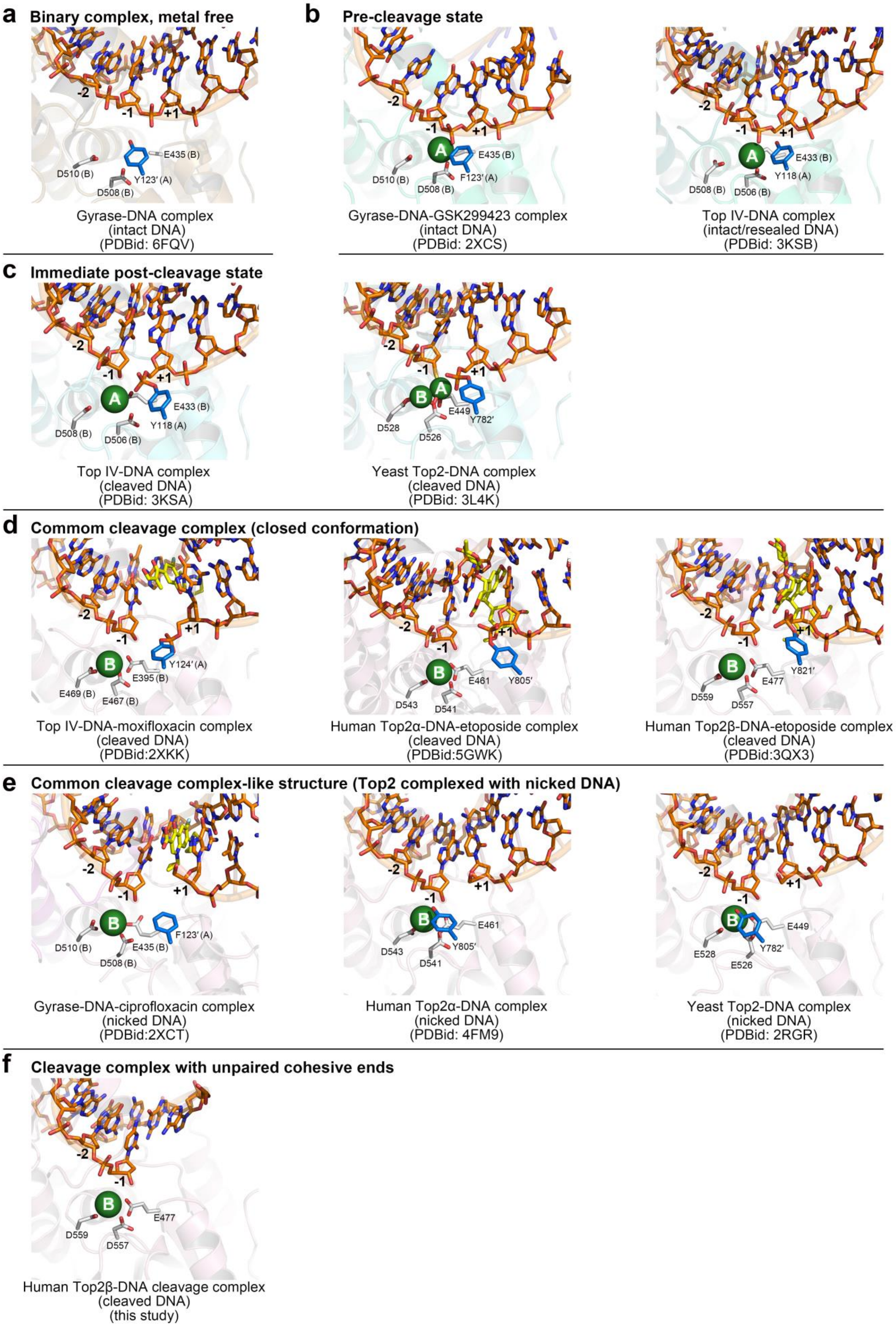
a Binary complex, metal free
-2
-1
+1
E435 (B)
D510 (B)
Y123′ (A)
D508 (B)
Gyrase-DNA complex
(intact DNA)
(PDBid: 6FQV)
b Pre-cleavage state
-2
-1
+1
A
E435 (B)
D510 (B)
F123′ (A)
D508 (B)
Gyrase-DNA-GSK299423 complex
(intact DNA)
(PDBid: 2XCS)
-2
-1
+1
A
E433 (B)
Y118 (A)
D508 (B)
D506 (B)
Top IV-DNA complex
(intact/resealed DNA)
(PDBid: 3KSB)
c Immediate post-cleavage state
-2
-1
+1
A
E433 (B)
D508 (B)
Y118 (A)
D506 (B)
Top IV-DNA complex
(cleaved DNA)
(PDBid: 3KSA)
-2
-1
+1
B
A
E449
Y782′
D528
D526
Yeast Top2-DNA complex
(cleaved DNA)
(PDBid: 3L4K)
d Commom cleavage complex (closed conformation)
-2
-1
+1
B
Y124′ (A)
E395 (B)
E469 (B)
E467 (B)
Top IV-DNA-moxifloxacin complex
(cleaved DNA)
(PDBid:2XKK)
-2
-1
+1
B
E461
Y805′
D543
D541
Human Top2α-DNA-etoposide complex
(cleaved DNA)
(PDBid:5GWK)
-2
-1
+1
B
Y821′
E477
D559
D557
Human Top2β-DNA-etoposide complex
(cleaved DNA)
(PDBid:3QX3)
e Common cleavage complex-like structure (Top2 complexed with nicked DNA)
-2
-1
+1
B
F123′ (A)
D510 (B)
E435 (B)
D508 (B)
Gyrase-DNA-ciprofloxacin complex
(nicked DNA)
(PDBid:2XCT)
-2
-1
+1
B
E461
D543
Y805′
D541
Human Top2α-DNA complex
(nicked DNA)
(PDBid: 4FM9)
-2
-1
+1
B
E449
E528
Y782′
E526
Yeast Top2-DNA complex
(nicked DNA)
(PDBid: 2RGR)
f Cleavage complex with unpaired cohesive ends
-2
-1
B
E477
D559
D557
Human Top2β-DNA cleavage complex
(cleaved DNA)
(this study)

Supplementary Figure 3. Based on the integrity of the G-segment, occupancy of the two divalent metal ion binding sites, and the formation of the phosphotyrosyl bond, the structures of the DNA-Top2 complexes can be grouped into six classes that may represent snapshots of Top2 at different catalytic stages during the DNA cleavage reaction. (**a**) **Binary complex, metal free**: this conformation (PDBid: 6FQV[2]) likely represents the initial association between Top2 and the G-segment. Compared to the other states, the metal-coordinating DxD motif of the TOPRIM domain is more distant from the DNA, suggesting that the TOPRIM domain has not yet fully engaged in DNA-binding. (**b**) **Pre-cleavage state**: in this conformation (PDBid: 2XCS[3] and 3KSB[4]), the metal ion bound in the A-site is jointly coordinated by the DxD motif and the scissile phosphate, an arrangement suitable for stabilizing the transition state arises during DNA cleavage. The catalytic tyrosine (or the phenyalanine from the catalytically dead mutant) appears ready for initiating the transesterification reaction by pointing directly at the scissile phosphate. (**c**) **Immediate post-cleavage complex**: this conformation (PDBid: 3KSA[4] and 3L4K[5]) is thought to occur right after the formation of the phosphotyrosyl linkage, in which the scissile phosphate and the DxD motif remain in close proximity to each other, and the A-site is shown to be occupied by a metal ion. Note that the B-site is also occupied when Zn^{2+} was used in place of Mg^{2+} for the preparation of cleavage complex (PDBid: 3L4K[5]). (**d**) **Common cleavage complex (closed conformation)**: in the structures belonging to this class (PDBid: 2XKK[6], 5GWK[7] and 3QX3[1]), the +1 and -1 nucleotides move slightly away from each other, which uncouples the scissile phosphate from the DxD motif. The structural integrity of the A-site is thus disrupted and a single metal ion is observed to occupy the B-sites. (**e**) **Common cleavage complex-like structure (Top2 complexed with nicked DNA)**: in these structures (PDBid: 2XCT[3], 4FM9[8] and 2RGR[9]), the spacing between the +1 and -1 nucleotides is similar to that seen in panel *d*, and only the B-site is occupied by metal ion. (**f**) **Cleavage complex with unpaired cohesive ends**: the observation of a metal ion bound in the B-site in the newly determined structure (PDBid: 5ZEN, this study) suggests that the structural integrity of the B-site and the presence of the B-site metal ion are not affected by the opening of the DNA-gate.

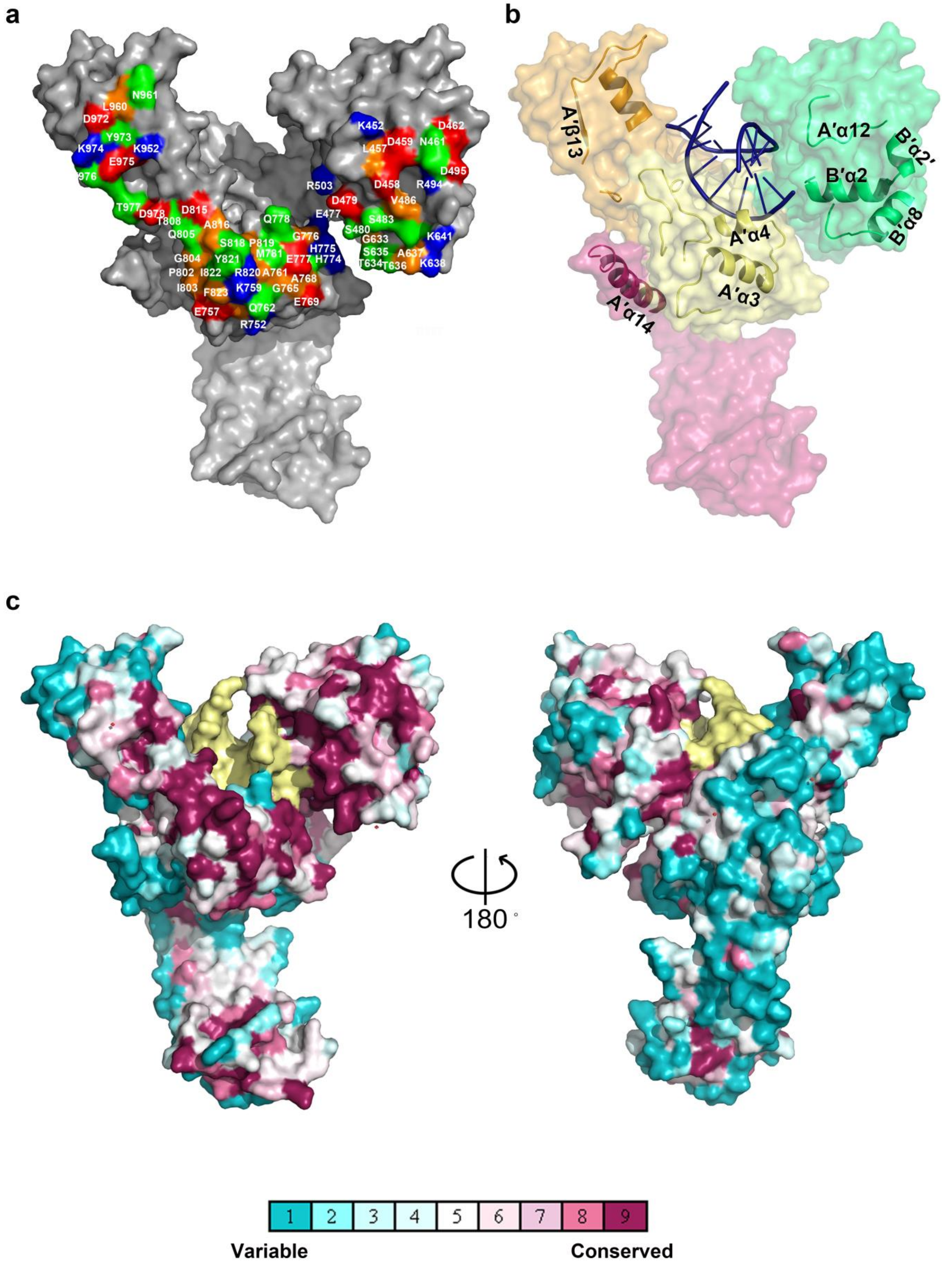

a
b
c
N961
L960
D972
Y973
K974
K952
E975
976
T977
D978
T808
D815
A816
Q805
S818
P819
Q778
G804
Y821
M781
E777
G776
P802
I822
R820
A761
A768
I803
K759
G765
F823
E769
E757
Q762
R752
K452
D462
D459
N461
L457
D495
R503
D458
R494
V486
D479
E477
S483
S480
H775
H774
G633
S635
T634
T636
A637
K641
K638
A′β13
A′α12
B′α2′
B′α2
B′α8
A′α4
A′α3
A′α14
180 °
1
2
3
4
5
6
7
8
9
Variable
Conserved

Supplementary Figure 4. Surface features of the T-segment-conducting path. (**a** and **b**) Amino acid residues and secondary structural elements lining the T-segment-conducting path are indicated. Residues are colored according to the following scheme: basic-blue; acidic-red; polar-green; and hydrophobic-orange. The TOPRIM, WHD, tower and coiled-coil/C-gate domains are shown in green, yellow, orange, and purple, respectively. (**c**). Conservation analysis of the surface residues. This figure was generated using the ConSurf server (http://consurf.tau.ac.il/). Each surface residue is colored based on the calculated degree of conservation according to the nine-color conservation scores shown at the bottom.

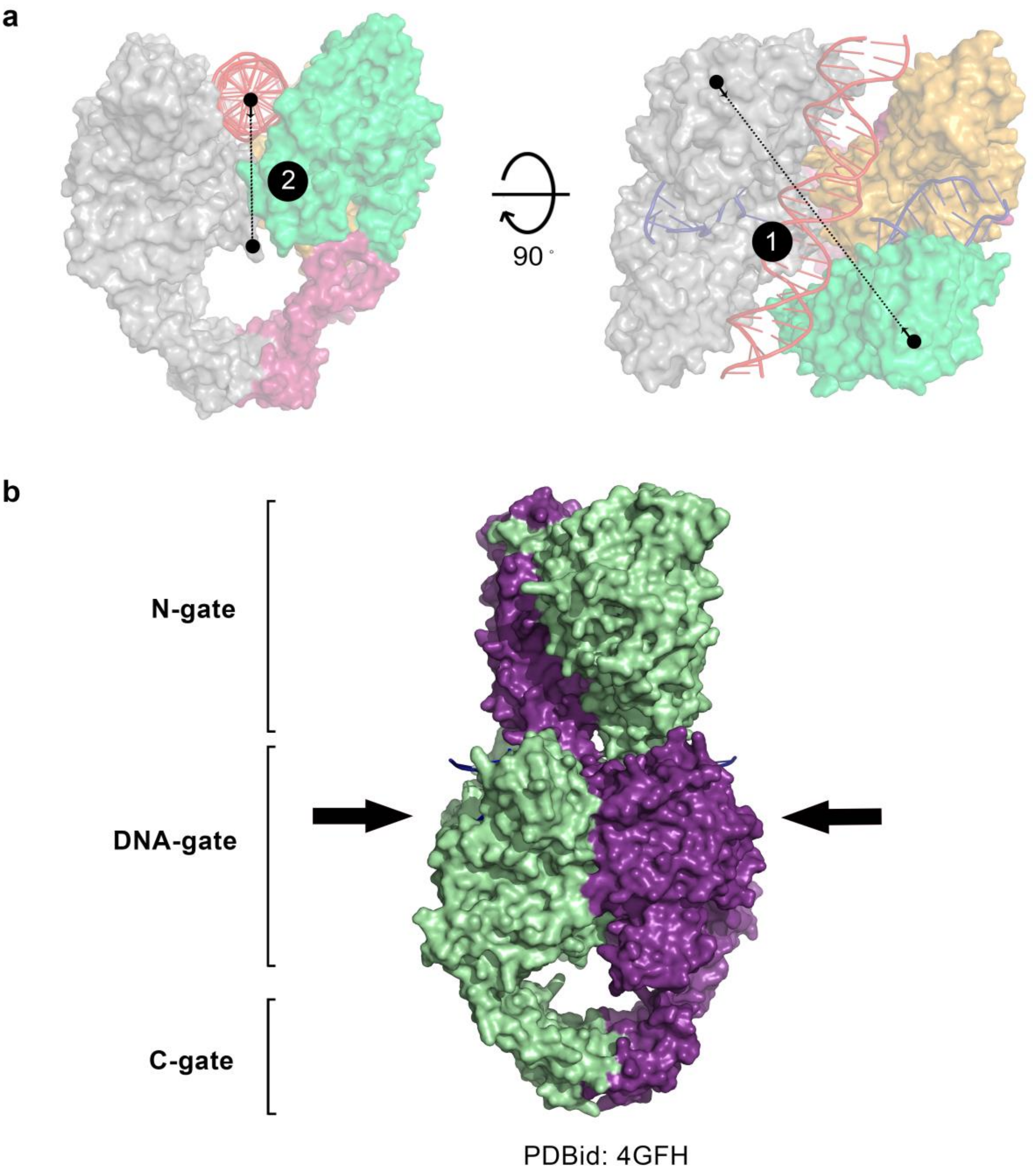

Supplementary Figure 5. A schematic diagram illustrating the strategy used for conducting the steered molecular dynamics simulations. Steered MD simulations of the strand passage process were conducted by introducing two virtual springs with a fixed force constant to control the movements of the tethered parts of the complex over time. (**a**) One of the springs was used to bring the T-segment from the top of the G-segment closer to the base of the WHD domains; the other was placed between the centroids of the TOPRIM domains on each side of the complex to bring them closer. (**b**) Crystal structure of AMPPNP-bound full-length *Saccharomyces cerevisiae* Top2[10] (PDBid: 4GFH) reveals that dimerization of the N-gate may induce a twisting motion and thus pull the two TOPRIM domains toward each other through a transducer linker, which may serve as the driving force for pushing the T-segment across the DNA-gate.

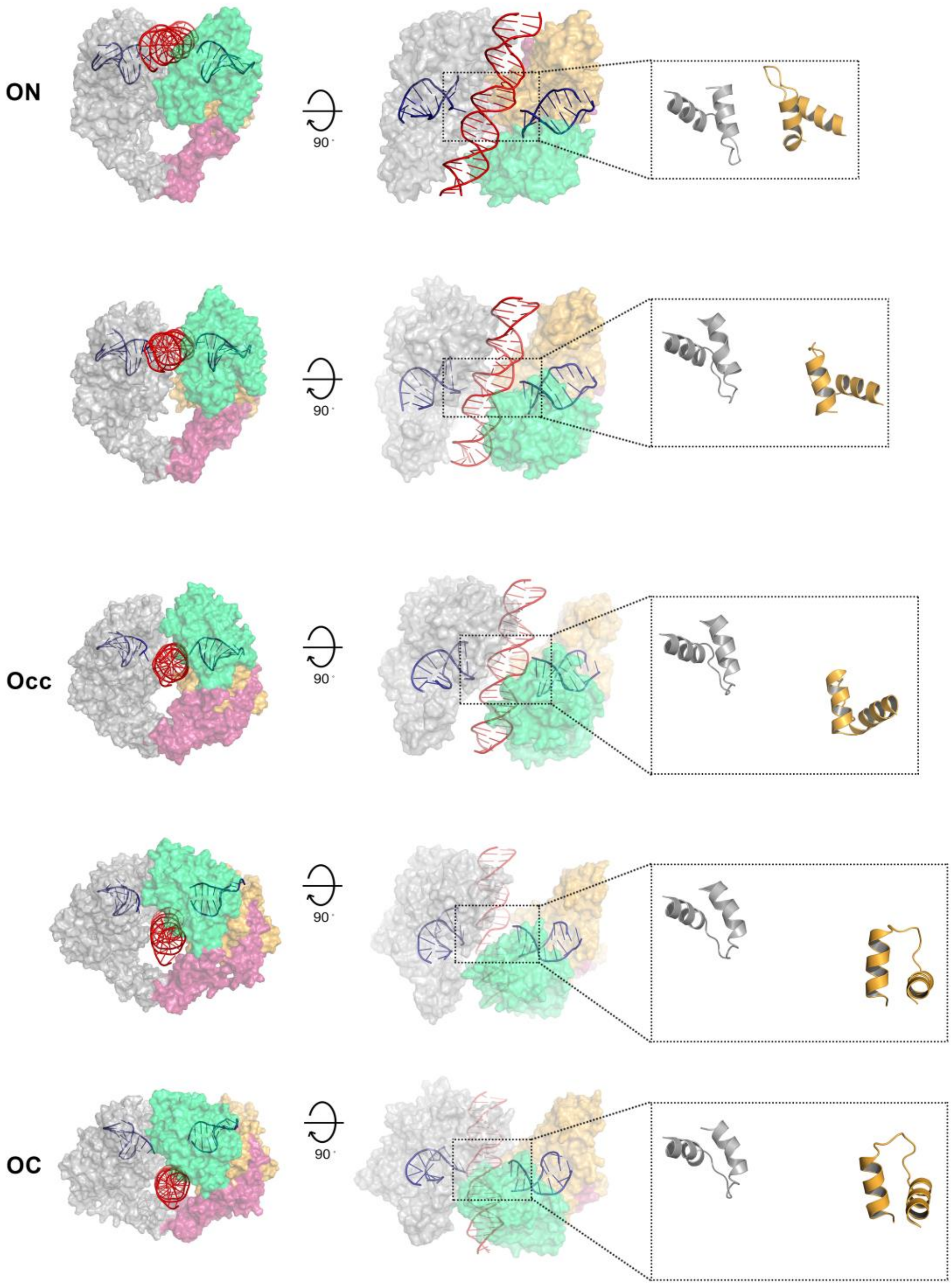

Supplementary Figure 6. Five snapshots taken from the steered MD simulation, which started from the ON (open and N-gate-facing) state through an apparent Occ (occluded) state and ended at the OC (open and C-gate-facing) state, revealing the "rocker-switch"-type conformational change of the DNA-gate during the simulated strand passage process. Notably, top views (down the molecular dyad of the Top2 homodimer) show that the T-segment undergoes an ~30° counterclockwise rotation as it crosses the DNA-gate. Also note that the simulated opening of the lower part of the DNA-gate appears to follow the sliding mode identified in Fig 3. The protein and DNA (G-segment in blue; T-segment in red) are shown in surface and cartoon representations, respectively.

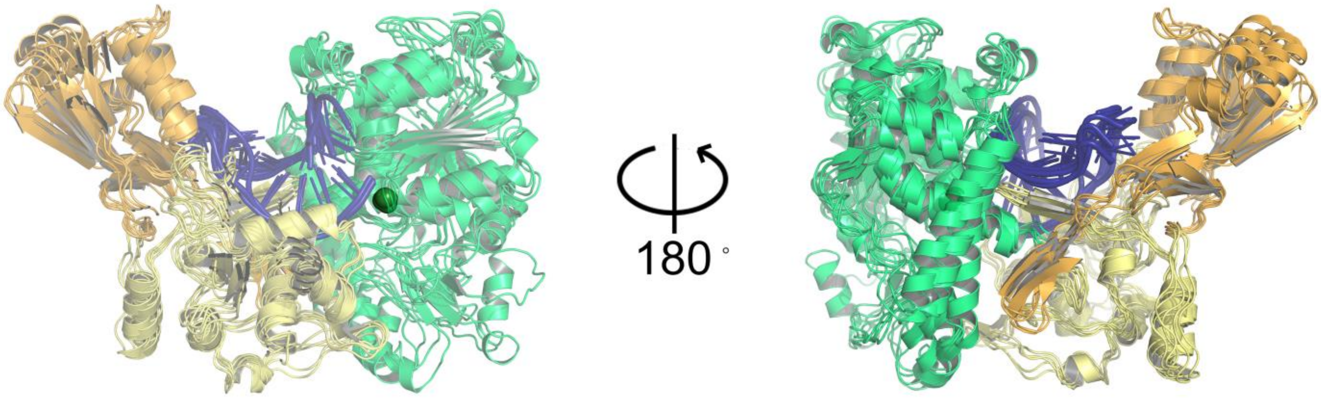

Supplementary Figure 7. The structural alignment of five selected intermediate structures (including the ON, Occ and OC states) that occurred during simulation revealed that the intricate interplays between the various protein domains (WHD (yellow), tower (orange), and TOPRIM (green)) and the cleaved G-segment (blue) are well preserved. Note that a divalent metal ion (green sphere, left panel) is faithfully retained at the B-site in all these structures.

a

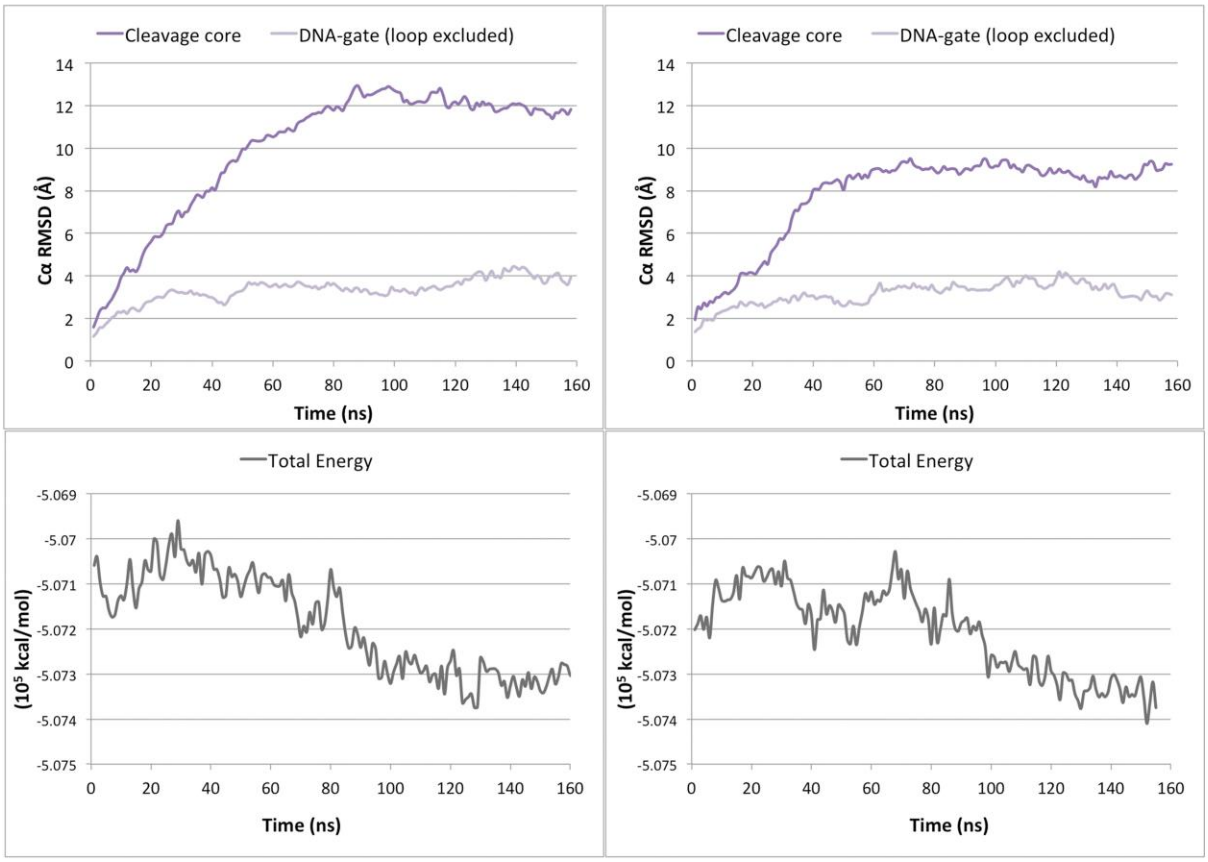

b

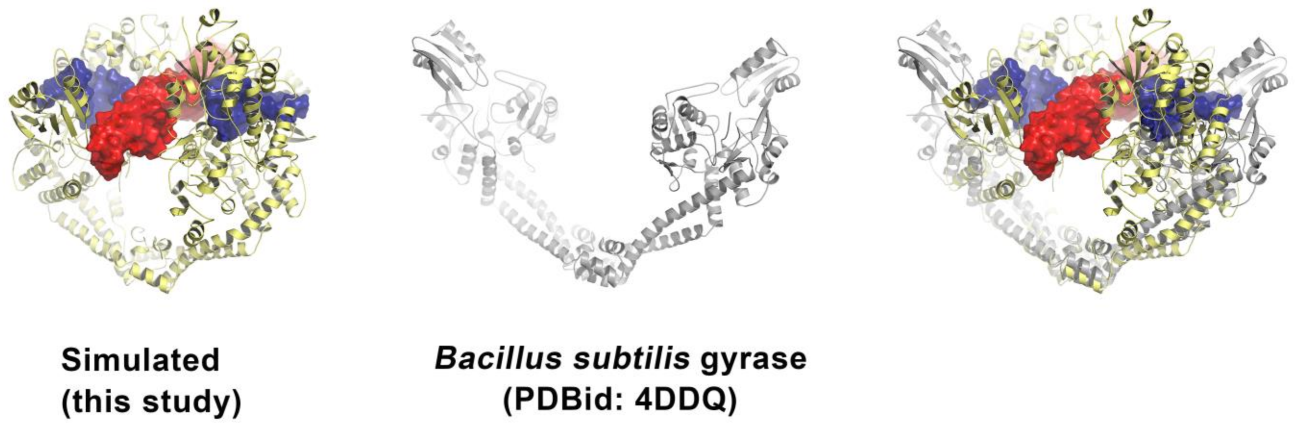

Supplementary Figure 8. Steered molecular dynamics simulations reveal a common structural snapshot adopted by the hTop2β and the Bacillus gyrase. (**a**) We conducted test steering simulations by gradually changing the rate of strand passage from 10-ang/ns to 0.25-ang/ns. These simulations showed similar structural changes of the central cavity and movement of the DNA gate. Two repetitions of the 0.25-ang/ns simulations were used in subsequent analyses for inspection, Simulations I (left) and II (right). Top: RMSD, relative to the starting structure, of alpha carbons of the entire cleavage core (dark curve), and of the DNA-gate component of the core excluding its loop region (pale curve), as a function of time, as the T-DNA migrates through the DNA gate. In each time window, the indicated component of the protein was optimally superposed on its

starting structure before the RMSD was computed. For each curve, the RMSD of each protomer was calculated separately, and the curves report the average over the two protomers. The modest elevation of RMSD for the overlaid DNA gates, relative to that of the complete protomer, indicates that most of the structural change during the 50-80 ns period result from shifts in relative position and orientation of the domains, leading to a structural change of the central cavity, rather than from conformational change in the DNA gate itself. Bottom: a steady decrease in the total energy of the two simulations is apparent in both runs. (**b**) A simulated intermediate structure of the Top2 cleavage complex that occurs during the T-segment gating resembles the conformation observed for the apo, DNA-free gyrase A subunit from *Bacillus subtilis* (pink; PDB code 4DDQ[11]). Because the TOPRIM domain of gyrase is part of the B subunit and thus absent in the Bacillus structure, the TOPRIM domains of the simulated structural intermediate were excluded from the superposition (right).

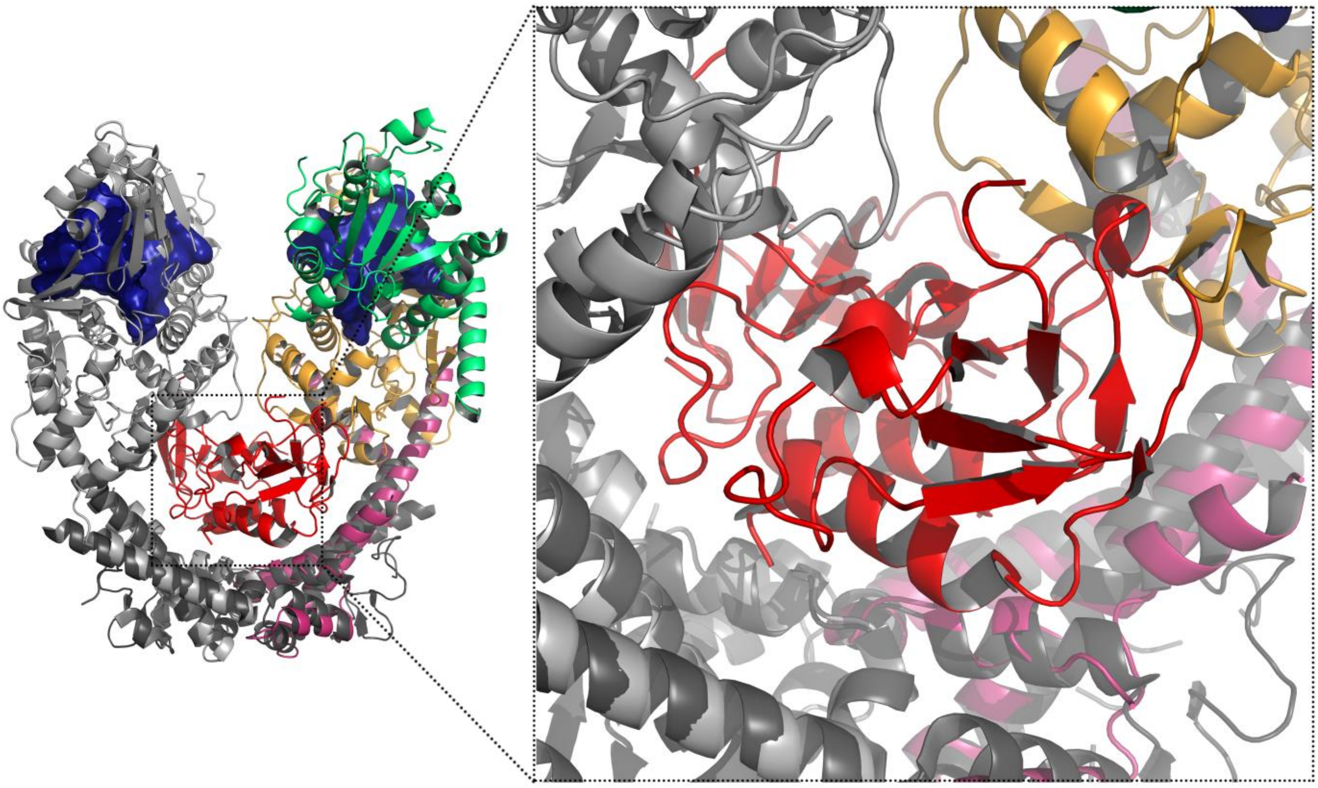

Supplementary Figure 9. The newly observed conformational state of Top2 may be targeted for the production of DNA double-strand breaks. The bacterial toxin CcdB is known to induce the formation of DNA double-strand breaks by targeting the C-gate region of gyrase (a bacterial Top2)[12]. Superimposing the crystal structure of the CcdB complexed with the gyrase C-gate fragment (PDBid: 1X75[12], pink) on the corresponding C-gate region in our newly determined structure (gray) indicates that the CcdB (red) can fit snugly within the cavity enclosed by the C-gate and DNA-gate. This modeling analysis implicates that CcdB may target the new Top2 conformation observed in this study.

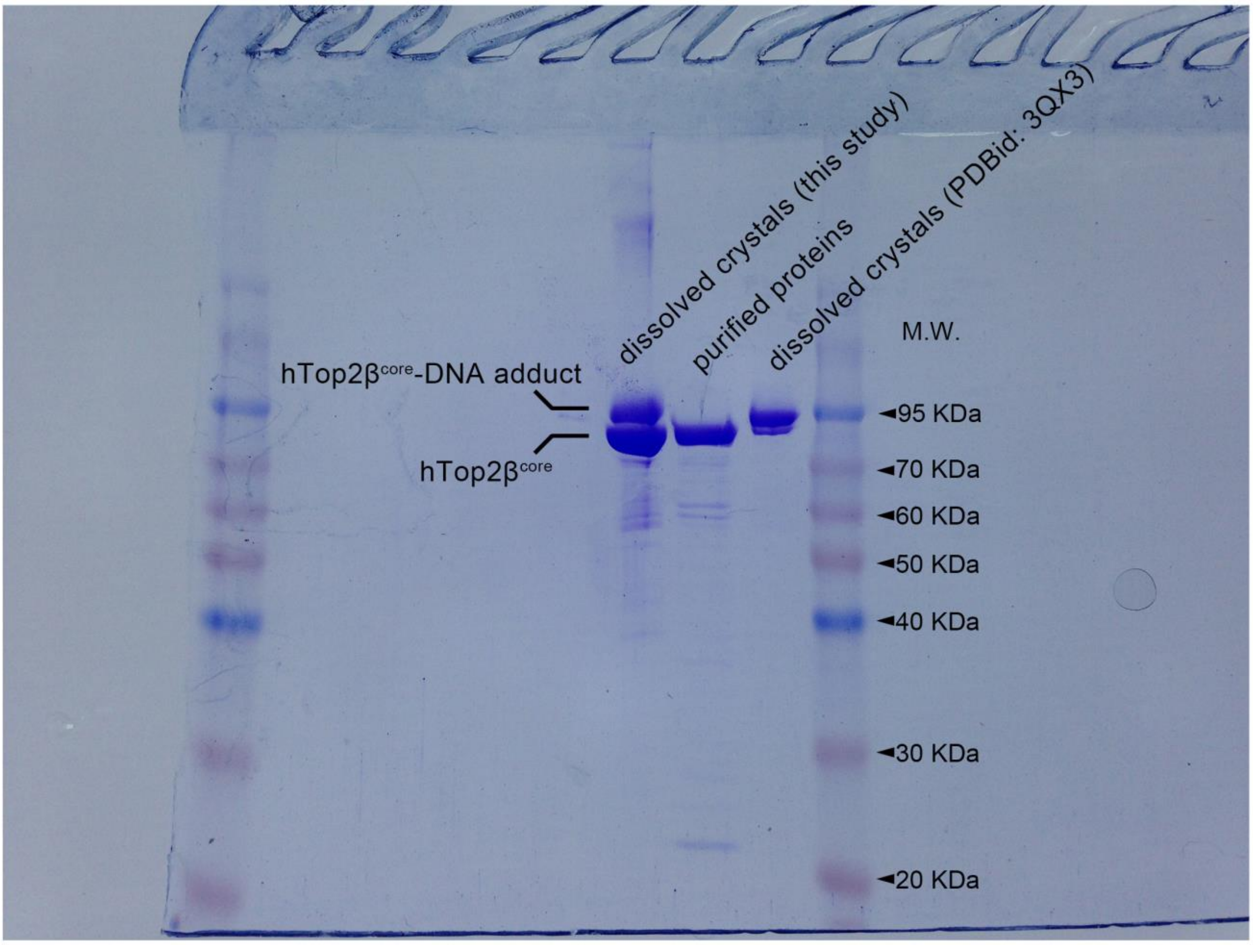

Supplementary Figure 10. SDS-PAGE analysis of the dissolved hTop2β^{core}-DNA crystals. Note that this is the uncropped image of Fig. 2d. The presence of a species that migrates slower than the apo (DNA-free) Top2 but similar to the Top2-DNA adduct suggests that the newly obtained crystal is composed of the Top2 cleavage complex.

Supplementary references:

1. Wu, C.C. *et al.* Structural basis of type II topoisomerase inhibition by the anticancer drug etoposide. *Science* **333**, 459-462 (2011).
2. Germe, T. *et al.* A new class of antibacterials, the imidazopyrazinones, reveal structural transitions involved in DNA gyrase poisoning and mechanisms of resistance. *Nucleic Acids Research* **46**, 4325 (2018).
3. Bax, B.D. *et al.* Type IIA topoisomerase inhibition by a new class of antibacterial agents. *Nature* **466**, 935-940 (2010).
4. Laponogov, I. *et al.* Structural basis of gate-DNA breakage and resealing by type II topoisomerases. *PloS one* **5**, e11338 (2010).
5. Schmidt, B.H., Burgin, A.B., Deweese, J.E., Osheroff, N. & Berger, J.M. A novel and unified two-metal mechanism for DNA cleavage by type II and IA topoisomerases. *Nature* **465**, 641-644 (2010).
6. Wohlkonig, A. *et al.* Structural basis of quinolone inhibition of type IIA topoisomerases and target-mediated resistance. *Nat. Struct. Mol. Biol.* **17**, 1152-1153 (2010).
7. Wang, Y.R. *et al.* Producing irreversible topoisomerase II-mediated DNA breaks by site-specific Pt(II)-methionine coordination chemistry. *Nucleic Acids Research* **45**, 10861-10871 (2017).
8. Wendorff, T.J., Schmidt, B.H., Heslop, P., Austin, C.A. & Berger, J.M. The structure of DNA-bound human topoisomerase II alpha: conformational mechanisms for coordinating inter-subunit interactions with DNA cleavage. *J. Mol. Biol.* **424**, 109-124 (2012).
9. Dong, K.C. & Berger, J.M. Structural basis for gate-DNA recognition and bending by type IIA topoisomerases. *Nature* **450**, 1201-1205 (2007).
10. Schmidt, B.H., Osheroff, N. & Berger, J.M. Structure of a topoisomerase II-DNA-nucleotide complex reveals a new control mechanism for ATPase activity. *Nat. Struct. Mol. Biol.* **19**, 1147-1154 (2012).
11. Rudolph, M.G. & Klostermeier, D. Mapping the spectrum of conformational states of the DNA- and C-gates in Bacillus subtilis gyrase. *J. Mol. Biol.* **425**, 2632-2640 (2013).
12. Dao-Thi, M.H. *et al.* Molecular basis of gyrase poisoning by the addiction toxin CcdB. *J. Mol. Biol.* **348**, 1091-1102 (2005).
