# Supplementary material for: Structural insights into the gating of DNA passage by the topoisomerase II DNA-gate
Source: Nat Commun. 2018 Aug 6;9:3085. doi: 10.1038/s41467-018-05406-y (PMC6078968; doi:10.1038/s41467-018-05406-y)
Supplement: Supplementary file 3 — Description of Additional Supplementary Files [file 41467_2018_5406_MOESM3_ESM.pdf]

Description of Additional Supplementary Files

File Name: Supplementary Movie 1
Description: Steered MD simulation of the full process of T-segment passage through the DNA-gate.
